# How Much Should We Weigh for a Long and Healthy Life Span? The Need to Reconcile Caloric Restriction versus Longevity with Body Mass Index versus Mortality Data

**DOI:** 10.3389/fendo.2014.00121

**Published:** 2014-07-30

**Authors:** Antonello Lorenzini

**Affiliations:** ^1^Department of Biomedical and Neuromotor Sciences, University of Bologna, Bologna, Italy

**Keywords:** longevity, life span, caloric restriction, dietary restriction, body weight, body mass index, mortality, obesity paradox

## Abstract

Total caloric restriction (CR) without malnutrition is a well-established experimental approach to extend life span in laboratory animals. Although CR in humans is capable of shifting several endocrinological parameters, it is not clear where the minimum inflection point of the U-shaped curve linking body mass index (BMI) with all-cause mortality lies. The exact trend of this curve, when used for planning preventive strategies for public health is of extreme importance. Normal BMI ranges from 18.5 to 24.9; many epidemiological studies show an inverse relationship between mortality and BMI inside the normal BMI range. Other studies show that the lowest mortality in the entire range of BMI is obtained in the overweight range (25–29.9). Reconciling the extension of life span in laboratory animals by experimental CR with the BMI–mortality curve of human epidemiology is not trivial. In fact, one interpretation is that the CR data are identifying a known: “excess fat is deleterious for health”; although a second interpretation may be that: “additional leanness from a normal body weight may add health and life span delaying the process of aging.” This short review hope to start a discussion aimed at finding the widest consensus on which weight range should be considered the “healthiest” for our species, contributing in this way to the picture of what is the correct life style for a long and healthy life span.

## Introduction

That moderation is a wise choice if we are concerned with health is an idea that has been handed down across cultures. The Japanese philosopher Kaibara Ekiken wrote about diet, advocating for moderation in food intake for better health as early as 1713. Scientifically, the first report on the health benefit of CR dates back to a study by McCay and colleagues in 1935 (1). In their report, extreme restriction in food intake retarded development in rats such that rats fed a restricted diet never achieved the adult weight of their unrestricted counterparts. Although smaller, these animals were longer lived. The life-prolonging effect of CR is more profound when restriction is started soon after weaning and carried out for the entire life of the animal. Nonetheless, if started in adulthood, so as not to effect development, the effect of CR is smaller but still highly significant (2). Life extension by CR seems universal, much less clear is the magnitude of life extension that varies widely among different orders (3). Recently, using a set of 41 recombinant inbred mouse strains originally developed for alcoholism research, Liao et al. (4) have challenged the universality of CR even in mice, which together with rats are by far the mammals more utilized in CR studies. A more universal phenomenon is the decrease in body weight that is associated with CR. When CR is started early after development, weight of CR mice ranges from ~60 to ~85% of the *ad libitum* weight (5). Weight loss is the result of both lean and fat mass loss, although variation in fat loss is the component mostly responsible for the high variation observed in weight loss among different strains (6). Humans are not different. If an adult will choose to calorically restrict its own diet, leaving everything else in its life style unchanged, the more consistent observable result will be a decrease in body weight.

**Figure 1:**
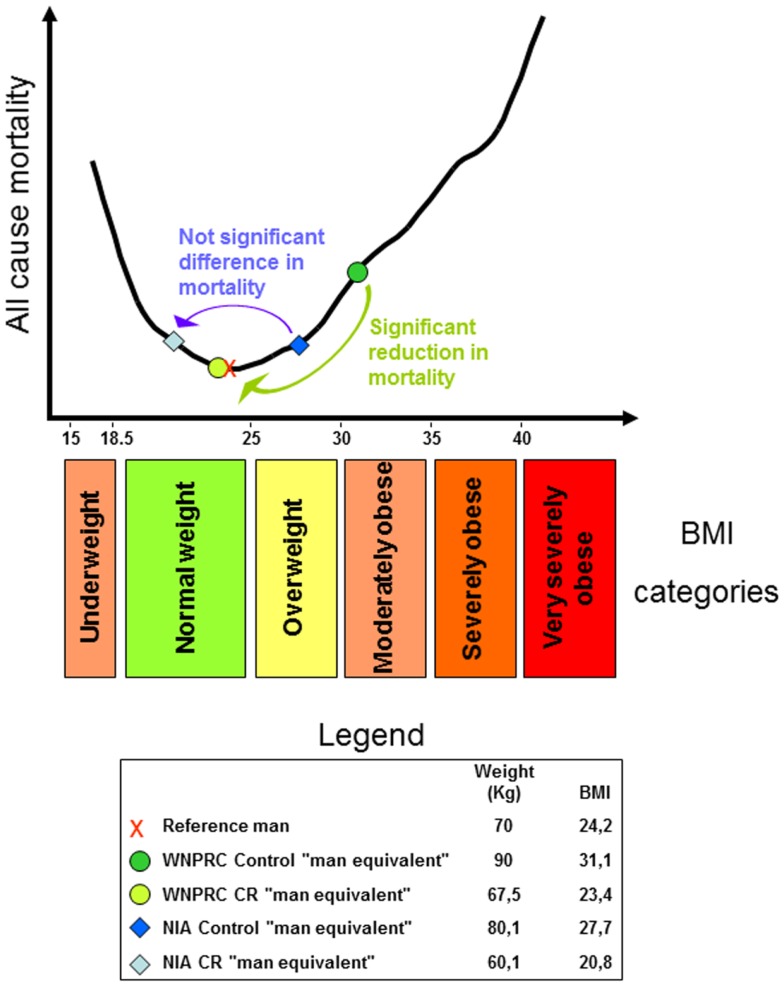
**U-shaped curve showing the relationship between all-cause mortality and body mass index (BMI) in man**. The curve was drawn using data from the Prospective Studies Collaboration et al. (9). For an explanation regarding the legend box and colored arrows, see the last paragraph.

The relationship between adult body weight and health in human is of extreme importance in our contemporary world. At present, a rough estimate of the “globesity” epidemic suggests that about one of every seven people is obese, two are overweight, and one is suffering from undernutrition (often of micronutrients). The body mass index (BMI), or Quetelet index, named after its founder Adolphe Quételet (7), is the ratio of a person’s mass (kilogram) to height squared (meter square) and is a widely used parameter for determining human body shape. In analysis of epidemiological data, the relationship between all-cause mortality and BMI gives a U-shaped curve indicating that extreme leanness as well as obesity tends to associate with increased mortality (Figure 1). As previously suggested, mortality data are more easily interpreted when translated into years of life lost or gained (8) and increased mortality of course means shorter life span. This review will examine the knowledge gained from CR experiments and attempt to reconcile these data with information gained from epidemiological studies in humans.

The focus here will be exclusively on body weight and on its relation to optimal health. With a given body weight of course, other factors are potentially very relevant in modulating health and life span, for example, diet composition and physical activity levels [see e.g., Ref. (10, 11) respectively]. Factors, these two just mentioned, that can contribute to health and life span independently from body weight and that are also capable, however, of modulating body weight and body composition profoundly.

We will first examine growth and longevity studies followed by an examination of lean body mass and longevity in several species used for experimental studies such as rodents. This information will be related to CR studies in primates, and finally to epidemiological data in the human population.

## Growth and Longevity

### Growth and longevity in animals

Species with greater adult body mass tend to be longer lived than species with smaller adult body mass, more precisely, with every doubling of species body mass, there is, on average, a 16% increase in maximum species life span (12). Within a single species, instead, and inverse relationship exists between body weight and lifespan. Citing the title of a study published by Miller and colleagues on this subject, we can say that in outbred mice, “big mice die young,” or to be more specific, “early life body weight predicts longevity” (13). Although, in rodents, this relationship is not always clear (14–17), a large analysis of laboratory rats and mice used for research in the twentieth century confirms these conclusions showing a negative correlation between maximum mature weight and maximum longevity (18). In other words, inside a single species, development, the rate and/or the extent of it, seems to be inversely related to longevity. A familiar species in which we can observe this relationship rather well is one that has been shaped for generations by human selective breeding, dogs. Breeds that grow to considerable size, probably in part because of high IGF-1 levels (19), tend to have shorter lifespans. For example, the Saint Bernard has an average lifespan just above 8 years, whereas the much smaller Chihuahua has an average life span of more than 10 years (20). Additionally, there are many mutations in mice in which small size is associated with increased longevity [for a review, see Ref. (21)]. Ames Dwarf mice, for example, which are homozygous for a recessive mutation in the *Prop 1* gene that causes hypopituitarism, weigh about one-third that of wild type mice and show an average lifespan extension of ~50% for males and 60% for females (22). Regarding hypopituitarism, we should add for completeness, that ablation of pituitary hormones is able to increase the lifespan of laboratory mice significantly even after complete development has been reached (23). Reasoning on what might explain the existence of such a trade-off between size and longevity, we have proposed the idea that the availability of time is key assets during development. A slower development (that can result, although not necessarily, in smaller adult size) means, at the cellular level, more time to complete every cell cycle (and of course fewer overall cell cycles in the case of smaller adult size). This should allow more time for error proofing in DNA synthesis and for damage repair, preserving the genome for longer periods and possibly extending lifespan (24). This relationship also has been observed in mice selectively bred for differences in the rate of body weight gain (25). More recently, in the short lived Berlin Fat Mouse Inbred line 860, it has been observed a highly significant inverse correlation between daily body weight gain and lifespan (26). Additionally, the trade-off between growth rate and longevity has been examined through experimental manipulation in fish. In three-spined sticklebacks, exposure to different temperature or photoperiod deflected normal growth trajectories. This induced catch-up or slowed-down growth that led to a reduced or extended lifespan, respectively (27). Finally, in a comprehensive analysis that used the AnAge database (28), postnatal growth rate and adult life span of 204 mammalian species are shown to be inversely and statistically significantly related (29). The same study reports also a positive and statistically significant relationship between time to sexual maturity and adult life span among 606 mammalian species, finding that is underlying again the importance of time during development.

### Growth and longevity in humans

Humans seem to not escape this inverse relationship. For example, baseball players weighing 80 or more kilograms have an average lifespan of about 61 years, whereas those weighing 10 kg less have an average lifespan of about 66 years (30). The same relationship has been observed between different ethnic groups living in California, where smaller size was associated with longer lifespan (30). However, mutations that extend lifespan in mice may not have the same impact in humans. Humans with a mutated *PROP 1* gene (similar to the Ames Dwarf) described on the Croatian island of Krk exhibit seriously compromised development. Although these individuals can reach advanced age, extreme longevity has not been reported (31, 32). From other available human studies, it appears that dwarfism, despite a reduced cancer risk (33), does not associate with increased lifespan [reviewed in Ref. (34)]. Studies analyzing human growth complicate the picture, showing that stunted or reduced growth during childhood due to poor nutrition and/or a higher infection load is associated with a decrease in lifespan, not an increase (35, 36). Although longevity data from human dwarfism and child growth studies suggest caution in drawing general conclusions, one possible hypothesis might be drawn from the studies thus far reviewed: factors with a positive effect on development, such as GH/IGF-1 and thyroid hormones, might negatively affect survival later in life. This could be an example of antagonistic pleiotropy (37) with beneficial effects on fitness early during lifespan and negative effects later. To this consideration, we should add that women are in average smaller than man and generally longer lived [reviews in Ref. (38)]. That size might partially account for the gender longevity benefit has in fact already been proposed (39, 40). Additionally, centenarians tend to be shorter than non-centenarians at 30 years (41). Finally, nonagenarians with greater body weight tend to have skin fibroblasts with less residual proliferative capacity compared with their smaller contemporaries, suggesting that larger bodies use more cellular replicative capacity during development, leaving less replicative potential for tissue maintenance during adulthood (42). This last finding is more relevant for the present discussion if we consider that the major determinant for skin fibroblast replicative capacity among species is size and not longevity (43).

This short overview of the relation between growth and longevity is of course incomplete. For more in depth reviews on the correlates between aging phenotypes and cellular phenotypes with a specific emphasis on cell proliferation, see Ref. (44, 45).

## Leanness and Longevity

### Leanness and longevity in rodents

The development of adipose tissue may be dampened by either physical activity or CR. In fact, adipocyte hyperplasia is significantly reduced if weight gain of Wistar rats is controlled by forced swimming or CR. If treatment is terminated in adulthood (28 weeks) and these animals begin to eat the same amount of food as sedentary control animals fed *ad libitum* throughout life, they will indeed significantly increase in weight, but will be unable to reach the same body weight as the control group, even at the advanced age of 62 weeks. This is mainly due to the reduced cellularity of adipose tissue during development caused by either of the two treatments (46). Masoro, studying Fischer 344 rats, reported that CR reduces the number of fat cells in fat depots when started soon after weaning or in adult life and that the capacity of CR to modulate fat cell number is maintained through most of the lifespan (47), indeed, differently from it seems to be the case in man (see below), in rats adipocytes numbers keeps on increasing even during adult life in *ad libitum* fed animals, at least this is the case for the Fisher 344 and the Wistar strains (46). Teillet and colleagues investigated the effect of CR on a lean strain of rats (WAG/Rij) and have concluded that food restriction initiated in adults most efficiently increases survival in rodents with a high spontaneous food intake (i.e., the majority of laboratory strains), but has a minor effect on lean strains (48). Liao et al. (6), measuring fat loss in 41 recombinant inbred strain of mice where CR was started in early adulthood (2–5 months of age), have obtained the opposite result. Strains with the least reduction in fat were more likely to show life extension after CR while those with the greatest fat reduction were more likely to undergo to lifespan shortening. Rodents with specific mutations that affect the adipose tissue complicate even more the picture. Genetically obese ob/ob mice are extremely fat (67% of body weight is fat) and short lived. They have been calorically restricted by receiving an amount of food so that they maintained their weight at a similar level of *ad libitum* fed normal mice. Surprisingly, they resulted longer lived than *ad libitum* fed wild type mice, and had similar longevities of equally food-restricted wild type mice although still 48% of their body weight was fat (49). Genetically altered mice that lack white adipose tissue result short lived (50) or diseased (51), but mice with a mutation that ablates the insulin receptor in adipose tissue are extremely lean and live significantly longer than wild type controls (52). Summing up the reports here reviewed, it is difficult to indicate a clear relationship between leanness and longevity for rodents.

### Leanness and longevity in monkeys

Studies in primates, of course, are of extreme relevance for understanding human endocrinology. In free-ranging human fed rhesus monkeys of the Cayo Santiago island of the Caribbean Primate Research Center, it is possible to observe obesity with abdominal fat accumulation (53); this indicates that macaques may have the same natural propensity for central obesity as humans do. Two large CR studies are ongoing in rhesus monkeys; one at the Wisconsin National Primate Research Center (WNPRC) in Madison (54, 55) and one at the National Institutes on Aging (NIA) in Bethesda, MD, USA (56). The application of CR is quite different in the two studies. The WNPRC study is conceptually more similar to a rodent study of CR started in adulthood. Fully developed monkeys assigned to the CR group were allowed free access to food for 3–6 months to determine their individual *ad libitum* intake. The CR diet consisted of their *ad libitum* intake reduced by 30%. In the NIA study, the control group of monkeys, once adult received a diet that prevented the appearance of obesity; in other words, they were not fed *ad libitum* as in the majority of rodent studies and the WNPRC study. The NIA CR group received a 30% reduction in caloric intake of their obesity-preventing diet. The difference in diet protocol between the two studies had a significant impact on body weight. At 17 years, WNPRC males weighed about 12% more than corresponding NIA males. The difference for females was about 18%.

Even with some monkeys still alive in both studies, expected overall results have been published. In the WNPRC study where CR monkeys weighed about 25% less than controls, mainly due to a reduction in body fat (57), a significant increase in average and maximum lifespan is observed (55). In the NIA study where CR monkeys also weighed about 25% less than controls (58), no significant increase in average or maximum lifespan is expected (56).

### Leanness and longevity in human

In humans, the influence of body fat on health has been since long time under scrutiny. Thanks to the ease with which it can be calculated, BMI is one of the anthropometric measurements most commonly used in epidemiological studies to assess overall body fat. However, its limitations are well known. For example, the BMI of an athlete may be in the range of obesity even if the subject has an exceptionally low percentage of body fat, due to the weight of a large lean body mass. In other words, BMI gives a very imprecise estimate of a person’s physical activity level, the importance of which has been since quite some time recognized in contributing to a successful aging (59) and can indeed significantly increase survival (11); for a recent discussion on the limits of BMI, see Ahima and Lazar (60). Notwithstanding these limitations, for large cohorts, BMI gives a good representation of leanness or lack thereof in a population (61). A recent, very large meta-analysis has shaken the epidemiological community by showing that the lowest inflection point for the BMI–mortality curve (its nadir) lays in the overweight range (62). Discussion is ongoing among epidemiologists on this topic, some time referred to as the “obesity–mortality paradox.” There are several confounding factors, in fact, to consider: for example, smokers tend to weigh less but have higher mortality (63); some chronic diseases may induce weight loss; the frail elderly with higher risk of death may also experience weight loss, etc. For a recent detailed review on this point, see Fontana and Hu (64). The issues of determining which of the two categories, normal or overweight, have the lowest mortality is of course highly relevant for the present discussion. However, even if we disregard this issue and observe the shape of the mortality curves with a higher resolution of the BMI scale that the one offered by the standard BMI categories, the difficulty of reconciling CR animal data with human epidemiological data becomes apparent. Starting from what is well known, it is clear that being underweight is associated with increased mortality and consequently reduced life expectancy. For example, from survival curves of patients with anorexia nervosa, it can be calculated that a person suffering from this disorder since the age of 15 years will endure a life-shortening effect of 25 years (65). What is not clear is where exactly the nadir of the curve lays and which curve should be used as reference for policy making decisions regarding social health. Of course, there are several important factors to keep in mind. For example, the nadir differs based on age. Andres (66) reported a rise in BMI from 21.4 for people aged 20–29 years to 26.6 for those aged 60–69 years. In another study, Matsuo et al. (67) reported an ~two-point increase in BMI moving from the 40- to 59-year age group to the 60- to 79-year group in both men and women. Additionally, data showing that the nadir for women tends to be lower than that of men (68) suggest that BMI categories should be different according to gender. Finally, the use of different reference curves might be appropriate based on ethnicity [on this last point, see for example, the studies listed in Table 1 and Ref. (69)]. Notwithstanding these complications, from the epidemiological literature it appears that the nadir of the curve does not lay in the middle of the normal range, but tends to lay near its upper limit if not above it, as previously mentioned. See Table 1 for an incomplete list of studies in which the BMI–mortality curve rise descending the normal range from 24.9 to 18.5.

**Table 1:** **Studies showing increasing mortality with decreasing BMI inside the normal BMI range (18.5–24.9)**.

Study title	Size of population analyzed (millions)	Notes	Reference
Association of all-cause mortality with overweight and obesity using standard body mass index categories: a systematic review and meta-analysis	2.88	Meta-analysis of 97 studies	Flegal et al. (62)
Body mass index and mortality among 1.46 million white adults	1.46	Meta-analysis of 19 studies, non-Hispanic white participants	Berrington de Gonzalez et al. (70)
Body mass index and cause-specific mortality in 900 000 adults: collaborative analysis of 57 prospective studies	0.9	Meta-analysis of 57 studies, participants mainly of western Europe and North America	Prospective Studies Collaboration et al. (9)
Shape of the BMI–mortality association by cause of death, using generalized additive models: NHIS 1986–2006	0.26	Non-Hispanic white participants	Zajacova and Burgard (71)
Body mass index and mortality in China: a 15-year prospective study of 220,000 men	0.22	Chinese cohort	Chen et al. (72)
Body mass index and mortality: results of a cohort of 184,697 adults in Austria	0.18	Austrian cohort	Klenk et al. (73)
Body weight and mortality among men and women in China	0.17	Chinese cohort	Gu et al. (74)
BMI and all-cause mortality among Japanese older adults: findings from the Japan collaborative cohort study	0.027	Japanese cohort	Tamakoshi et al. (75)

An important question that gerontologists and epidemiologists should try to answer together is the following: “people who voluntarily choose a CR regimen and are already within a normal BMI range, let us say its upper half, are increasing their longevity or their mortality?” Indeed, when glancing on reports about members of the Calorie Restriction Society, or CRONies (Calorie Restriction with Optimal Nutrition) as they call themselves, we should consider their BMI. In one of the longest studies available, for example, where subject were monitored for a period of 6 years, 28 weight-stable CRONies had an average BMI of 19.7, and they were compared with 28 age-matched subjects on a typical western diet who had an average BMI of 25.6 and served as the control group (76). These two groups, for example, had BMI values that quite precisely spanned the normal BMI range. If these two groups of persons will keep their body weight constant for the future, what could we predict regarding their longevity? Using as guidance studies like the ones reported in Table 1, we should conclude that the control group should experience a lower mortality. Instead, using as guidance the generally accepted idea that CR extend laboratory animals life span together with the few available prospective studies where persons who were leaner in youth or in midlife resulted longer lived (77, 78), we should conclude that the CRONies will actually experience decrease mortality and extended longevity.

To complete this short overview on human leanness, we should mention that recent data on body fat cellularity indicate that humans may behave as rodents during development (i.e., more available calories – more adipocytes), but a little differently in adulthood. Spalding et al. (79) reported that ~10% of fat cells are renewed annually in adults at every BMI level in humans. However, the number of fat cells remains constant in adulthood in both lean and obese individuals, and obese individuals that undergo bariatric surgery lose a significant amount of weight and body fat, but not fat cell number.

## Wild and Hypothetical Considerations

### Wild considerations

Wild mice housed in the laboratory for four generations under *ad libitum* conditions eat less than control mice of several strains used in CR experiments (80) and when these wild mice are subjected to CR, they do not benefit from extended longevity (81). Wild rhesus monkeys in their habitat result lighter even then CR monkeys of the NIA and WNPRC studies described above (82) and at least for the WNPRC study, the difference in weight between the CR and *ad libitum* groups is more a result of weight gain in the control monkeys than weight loss in CR monkeys (83). Altmann and colleagues report that baboons with accessible abundance of food had in average a 23.2% of body fat while wild-feeding animals had only a scarce 1.9% (84). Young adult WNPRC Rhesus monkeys have average body fat above 10% already at the beginning of the study (85). The percent of body fat of these monkeys then quickly increases during their adult life; and this it does happen, although in a lesser degree, even in the CR animals; for the majority of their adult life, indeed, body fat appears to be around: 30% for control males, 20% for CR males, 20% for control females, and 13% for CR females (57).

These data suggest that laboratory strains of rodents have been inadvertently selected for higher caloric intake and consequently for higher percent of body fat (80). An interesting meta-analysis on the universality of CR among all tested species suggests that this husbandry selection bias could explain why the mot significant effects of CR are observed in models species: mice and rats, *Drosophila melanogaster, Caenorhabditis elegans*, and *Saccharomyces cerevisiae* (3). The sedentariness imposed by the close confinement of captivity also may contribute to a higher percent of body fat because energy expenditure may not be correctly balanced with food consumption; at least this is true in male rats (46, 86, 87). For monkeys, in addition to low energy expenditure, we should consider that in the WNPRC and NIA studies the animals are housed individually and in very small cages [in the WNPRC study, cage volume is of 0.66 m^3^ (88)]. Social isolation may overstimulate food consumption as a compensatory mechanism of the reward limbic system due to life conditions that can understandably carry intelligent animals toward depression. Indeed Luppino et al. (89), in a recent meta-analysis, conclude that obesity and depression are reciprocally predictive. Finally, in northern Tanzania, the Hadza, who are one of the last remaining human hunting and gathering communities of the world have an average BMI of 20.2 for man and 20.4 for woman (90).

### Hypothetical considerations

Considering all of the above and adding that WNPRC male control monkeys weigh about 9% more than the average of captive monkey housed in research facilities across the US [see Ref. (55); and the iPAD database http://ipad.primate.wisc.edu], we may consider a control monkey of the WNPRC study as equivalent to a moderately obese man. Then, using the available data from the monkey studies [see Supplementary Table 2 of (56) and reference there in], propose the following speculation. Consider a 1.7 m tall reference man weighing 70 kg (91) with a BMI of 24.2 (in the upper half of the normal range). Then, imagine him obese, for example, 90 kg with a BMI of 31.1. We can use this man as the “man equivalent” of the WNPRC control monkey. The WNPRC CR “man equivalent” will weigh 25% less and consequently, will have a BMI of 23.4 (still in the upper half of the normal weight category). WNPRC male monkeys weighed ~12% more than corresponding NIA males so the control “man equivalent” of the NIA study will have a BMI of 27.7 and be in the overweight category. CR in the NIA study reduces body weight by 25%, so the NIA CR “man equivalent” will have a BMI of 20.8 (in the lower half of the normal weight category). Recapping and considering the recent literature on the relation between mortality and human BMI, it is possible to suggest that the WNPRC study showed a significant health advantage of the CR monkeys as a significant advantage can be effectively observed between obesity and normal weight in all-cause mortality in the vast majority of epidemiological human studies. On the other end, CR on the NIA monkeys fails to show a clear life span benefit as several human studies fails to show a clear advantage between the overweight category and the first half of the normal weight range (see an explicative scheme of this argument in Figure 1). In pondering this hypothetical parallel between humans and Rhesus monkey, let us consider also that only 10% of CR is equivalent to a restriction of 25% in increasing rats’ life span (92).

Of course, this is just one possible explanation in trying to reconcile animal data on CR and human epidemiological studies. It is an explanation that brings us back to a not new consideration: the one that caged animals with *ad libitum* feeding should be considered to be in an “obesogenic” instead of in a “normal” or “control” condition (93), a concept more recently reviewed by Martin et al. (94). The hope of this short review is that more in depth analysis will follow on this important matter. To better interpret animal CR studies, for instance, it could be useful to generate (for monkeys, for example, using resources such the iPAD just mentioned), mortality versus % body fat or mortality versus BMI curves for *ad libitum* fed control animals. Indeed, reference body composition values for adult rhesus monkeys have been already proposed (95).

## Conflict of Interest Statement

The author declares that the research was conducted in the absence of any commercial or financial relationships that could be construed as a potential conflict of interest.
